# Zinc finger proteins: guardians of genome stability

**DOI:** 10.3389/fcell.2024.1448789

**Published:** 2024-07-25

**Authors:** Zeeba Kamaliyan, Thomas L. Clarke

**Affiliations:** Department of Pathology and Laboratory Medicine, Boston University Chobanian and Avedisian School of Medicine, Boston, MA, United States

**Keywords:** zinc finger (ZNF), DNA repair, genome integrity, human disease, cancer

## Abstract

Zinc finger proteins (ZNF), a unique yet diverse group of proteins, play pivotal roles in fundamental cellular mechanisms including transcription regulation, chromatin remodeling, protein/RNA homeostasis, and DNA repair. Consequently, the mis regulation of ZNF proteins can result in a variety of human diseases, ranging from neurodevelopmental disorders to several cancers. Considering the promising results of DNA damage repair (DDR) inhibition in the clinic, as a therapeutic strategy for patients with homologous recombination (HR) deficiency, identifying other potential targetable DDR proteins as emerged vulnerabilities in resistant tumor cells is essential, especially when considering the burden of acquired drug resistance. Importantly, there are a growing number of studies identifying new ZNFs and revealing their significance in several DDR pathways, highlighting their great potential as new targets for DDR-inhibition therapy. Although, there are still many uncharacterized ZNF-containing proteins with unknown biological function. In this review, we highlight the major classes and observed biological functions of ZNF proteins in mammalian cells. We briefly introduce well-known and newly discovered ZNFs and describe their molecular roles and contributions to human health and disease, especially cancer. Finally, we discuss the significance of ZNFs in DNA repair mechanisms, their potential in cancer therapy and advances in exploiting ZNF proteins as future therapeutic targets for human disease.

## 1 Zinc finger proteins

**FIGURE 1:**
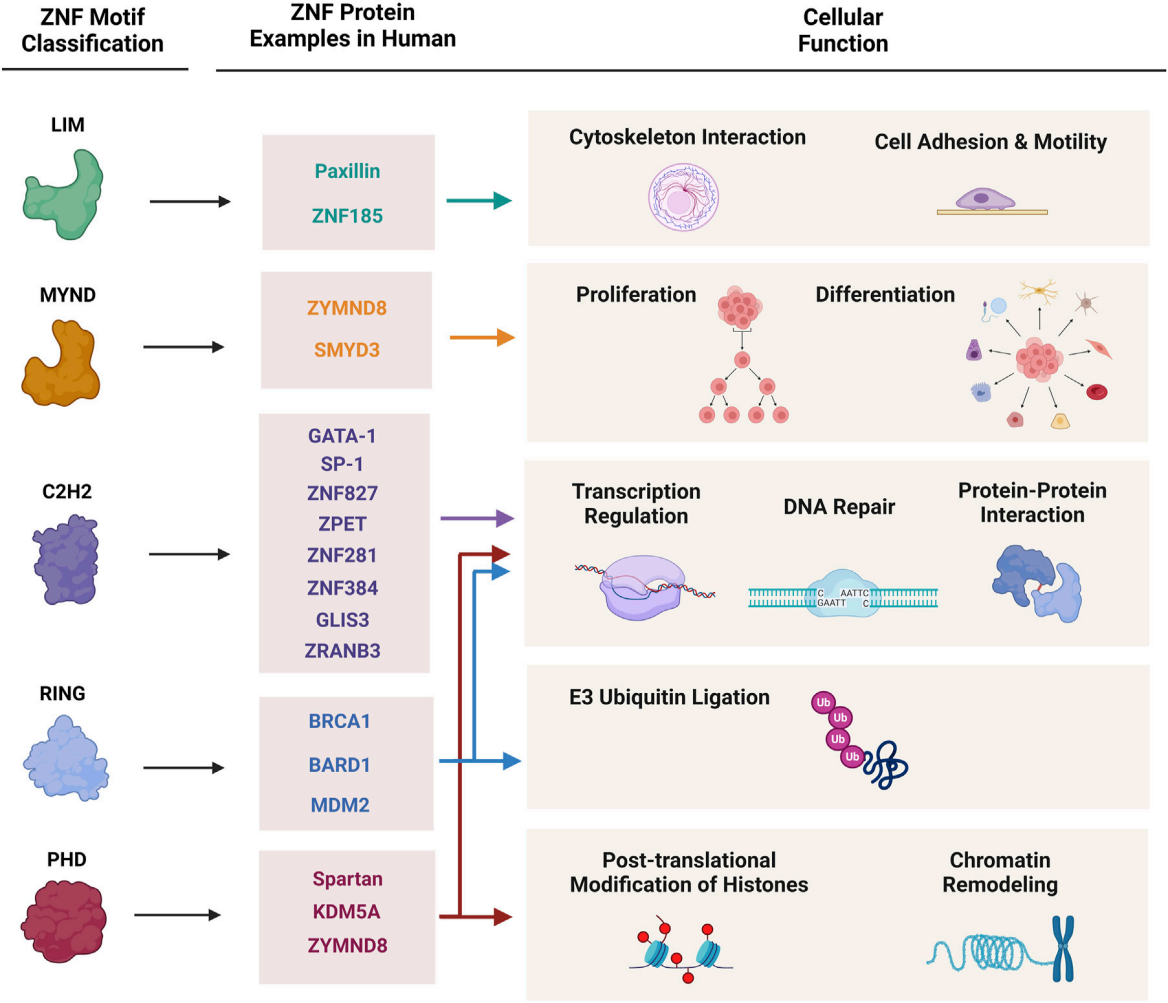
Classification of zinc finger proteins and their cellular functions. This figure demonstrates major classes of zinc finger proteins, characterized protein examples of each group, and their involvement in cellular and molecular mechanisms.

Zinc finger proteins (ZNFs) are the most abundant and diverse group of proteins which are encoded by 5% of the human genome. Zinc fingers are small motifs constituted of one or more zinc ions attaching to multiple Histidine and Cysteine amino acids in different arrangements (Vilas et al., 2018). The zinc ion in these fingers is a prerequisite to stabilize the conformation of ZNF proteins. This small particular structure gives ZNFs the stability and flexibility to bind and interact with different substrates including DNA, RNA, proteins, lipids, and act in different biological pathways (Padjasek et al., 2020). As shown in Figure 1, through their structural and functional diversity, ZNFs are involved in many fundamental cellular processes including transcription regulation, cell adhesion, protein degradation, DNA damage repair, chromatin remodeling, and more, consequently playing a critical role in human health and disease (Cassandri et al., 2017).

ZNF proteins are mainly classified according to their structural characteristics, zinc finger motif topologies and their intervening sequences, to multiple subgroups: C2H2, LIM, MYM, RING, and PHD (Matthews et al., 2009). Many ZNFs may contain multiple functional domains and therefore, play roles in different cellular pathways. On the other hand, there are some ZNFs with completely different structural domains that share a similar biological function in cells (Figure 1).

### 1.1 C2H2

This classical type of ZNF domain is consisted of 2 Cysteine and 2 Histidine residues associated with one zinc ion, which can bind to specific DNA sequences when arranged in tandem repeats. The C2H2 containing proteins comprises the largest group of zinc finger proteins in eukaryotes, many of which function as transcription factors (Zhang et al., 2011). The appealing feature of detecting and binding to selective and specific DNA base pairs have made them a potential genome editing tool (Wolfe et al., 2000). Although, there are C2H2 motifs with the ability for protein-RNA and protein-protein interactions too. GATA-1 is a well-known transcription factor belonging to this ZNF category, which has other functional domains too. It has been reported that GATA-1 can switch between its transcriptional regulation and DNA-binding function according to its interacting protein partner in the nucleus (Wolfe et al., 2000; Jen and Wang, 2016). Another notable C2H2-containing ZNF with dual roles in cellular mechanisms is the Sp1 transcription factor. Recent findings uncovered a previously unknown RNA-binding capability for Sp1, independent from its well-established DNA-binding and gene expression regulatory functions. This work demonstrated that Sp1 regulates alternative poly-adenylation (APA) and mRNA stability, proposing its role as a potential oncogene in breast cancer cells (Song et al., 2022).

### 1.2 LIM

LIM domain, named after three initially discovered proteins Lin-ll, Isl-1, and Mec-3, has two sequential zinc-binding units in linear pattern and show diverse topologies and functions including: expression regulation, cell adhesion, cell motility, cytoskeleton interaction, and more (Matthews et al., 2009). They are known as exclusive protein-recognition motifs and can act as molecular bridges (Gamsjaeger et al., 2006). Paxillin for instance, recruits to actin strain sites of the cell as an adaptor protein to gather other cytoskeleton regulators to promote actin recovery (Smith et al., 2013). ZNF185, a single LIM-containing protein shows greater association with actin-cytoskeleton and is enriched in focal adhesions in cells. Upregulation of ZNF185 has been reported to suppress cell proliferation and given its epigenetic silencing in different tumor cells, this protein has been proposed as a tumor suppressor (Zhang et al., 2007).

### 1.3 MYND

The MYND domain is similar to the LIM domain in many aspects including having two zinc-binding modules sequentially and also acting predominantly as a protein-biding domain (Gamsjaeger et al., 2006). Binding to Proline-rich protein counterparts can be their most specific feature. Also, many of MYND containing ZNFs contribute to developmental processes and consequently in cancer-related processes like proliferation and differentiation (Cassandri et al., 2017). Recently, a ZNF protein ZMYND8, has been identified to play a vital role in DNA damage repair (DDR) and genome stability of human cells as a chromatin reader protein. Studies have demonstrated that the MYND domain of ZYMND8 is crucial for recruitment of this protein to DNA damage sites facilitating DNA double-strand break (DSB) repair. Interestingly, ZYMND8 has also been reported to associate with other ZNFs: ZNF532, ZNF592, ZNF687 and PARP1 in DNA damage sites to promote the homologous recombination (HR) pathway (Spruijt et al., 2016; Vilas et al., 2018).

### 1.4 RING

The RING (really interesting new gene) domain of ZNFs is mostly present in E3 ubiquitin ligases. BRCA1 (breast cancer gene 1), BARD1 (BRCA1 associated RING domain 1) and MDM2 (mouse double minute 2 homolog) are well-known proteins containing this domain which play important roles in cancer progression (Cassandri et al., 2017). In most cases dimerization of the RING domain is necessary to bind and mark substrates. For example, BARD1 protein (BRCA1-associated RING domain 1) forms a heterodimer with BRCA1 to enhance its E3 ubiquitin ligase activity (Matthews et al., 2009). RING domain proteins can also contribute to the assembly of large protein complexes, therefore taking part in cellular functions like transcription regulation and DNA repair, which require cooperation between several large multi-protein complexes (Matthews and Sunde, 2002).

### 1.5 PHD

PHD-containing ZNFs are the classical chromatin structure remodelers that participate in epigenetic regulation. The PHD motif structure is so similar to that of the RING motif that they were initially considered as protein ubiquitin ligases too (Aravind et al., 2003; Matthews et al., 2009). Most of the PHD-domain members are involved in post-translational modifications (PTMs) of Histones and therefore, DNA structure-based processes such as DNA repair and chromatin regulation (Singh and van Attikum, 2021). ZYMND8 which has been mentioned above as a MYND-containing ZNF, has the triple reader-cassette module PHD/BRD/PWWP, bringing it to the chromatin remodeler category too. Interestingly, the histone demethylase KDM5A, containing three PHD domains, also interacts with ZYMND8 and is needed for the accumulation and engagement of this important chromatin reader to DNA damage sites (Vilas et al., 2018). These proteins are a good example of multi-functionality of ZNFs in DNA repair, protein modifications and chromatin stability.

## 2 Zinc finger proteins and DNA repair

Our genome is continuously exposed to exogenous (environmental) and endogenous sources of DNA damage. Therefore, multiple integrated DNA damage repair (DDR) pathways have developed to sense, detect and repair different types of DNA lesions, in different cellular stages (Ciccia and Elledge, 2010). Depending on the nature of DNA damage, specific response mechanisms can be triggered. Mismatch Repair (MMR) corrects mismatched base pairs in DNA strands, alterations in base structures are reversed mostly by base excision repair (BER), while more complex lesions involving multiple nucleotides are removed by nucleotide excision repair (NER). Single strand breaks (SSB) can be detected and repaired by single-strand break repair pathway factors, and double strand breaks (DSB) can be corrected either by homologous recombination repair (HR) or non-homologous end-joining (NHEJ) mechanisms (Dexheimer, 2013). DSB repair mechanisms are of prominent importance in the cell due to the highly toxic nature of DSBs to genome stability. If these lesions are not sensed or corrected properly, this can lead to a variety of human diseases including cancer, neurodegenerative disease and several developmental and immunodeficiency syndromes (Jackson and Bartek, 2009; Clarke and Mostoslavsky, 2022).

It has been shown that different DDR pathways can cooperate at the same time to edit a DNA lesion and there are some DNA repair proteins that function in more than one repair pathway, consequently playing a more prominent role in protecting our genome integrity (Jackson and Bartek, 2009). Interestingly, many of these multi-functional DDR proteins contain ZNF domains like PARP1, ATM, P53 and BRCA1 (Cassandri et al., 2017).

PARP1 (poly-ADP ribose polymerase 1) acts as a DNA damage sensor that is crucial to recruit DDR factors including chromatin remodelers, DNA repair proteins and transcription factors, via adding poly ADP-ribose chains to DNA break sites. This enzyme contains three specific DNA-binding zinc finger domains and has been shown to participate in multiple repair mechanisms including: single-strand break repair (SSBR), BER, HR, and NHEJ (van Beek et al., 2021). Studies have revealed that many ZNF proteins involved in DNA repair function in a PARP-binding-dependent manner, leading to the identification of the PAR-binding zinc finger module (PBZ) (Ahel et al., 2008).

## 3 PARPi

In recent years DDR inhibition has been identified as an effective strategy for cancer patients with specific DNA repair defects. PARP1 inhibitors, like Olaparib, are the best example of this treatment that has been relatively successful to treat breast, prostate, pancreatic and ovarian cancer patients carrying deleterious HR defects, like *BRCA1/2* mutations (Clarke and Mostoslavsky, 2022). Farmer et al. (2005) and Bryant et al. (2005) indicated for the first time that *BRCA1/2* mutations sensitize tumor cells to PARP inhibition due to the persistence of DSBs, resulting in genome instability and cell death. They identified that lack of PARP1 activity resulted in unrepaired single strand DNA breaks, which eventually resulted in DNA DSBs that needed to be repaired via homologous recombination. In addition to inhibiting the synthesis of poly PAR chains, PARPi drugs can also alter the conformation of this protein preventing its release from DNA, known as PARP1 trapping. This phenomenon induces DNA replication fork collapse and the persistence of unrepaired DNA damage during S phase, resulting in genomic instability and cell death in HR-deficient cells. Given that cells which harbor mutations in *BRCA1/2* are deficient in homologous recombination repair, these cancer cells are exquisitely sensitive to this treatment, a concept known as synthetic lethality (Bryant et al., 2005; Farmer et al., 2005; Li et al., 2021; Baxter et al., 2022). Following this promising therapeutic strategy, other DDR inhibitor drugs targeting other repair proteins like ATR (Ceralasertib), WEE1 (Adavosertib), CHK1 (Prexasertib), and POLθ (Novobiocin) have been developed and are currently undergoing testing in clinical trials (Zhou et al., 2021; Baxter et al., 2022).

## 4 PARPi resistance

Although initially promising, PARPi resistance have been observed routinely in the clinic after prolonged exposures. Multiple mechanisms have been reported which can compensate for HR defects (HRD) in tumor cells. For instance, reversion mutations that bring back the natural protein function has been reported in *BRCA2*, as well as *BRCA1*, *RAD51* and *PALB2*, in hundreds of patients treated with PARPi (Edwards et al., 2008). In cases of promotor hypermethylation, like *BRCA1* and *RAD51C* in some cancers, loss of methylation might also reverse the HRD phenotype (Baxter et al., 2022). Another mechanism is alterations in other DDR protein genes like 53BP1, which has been found in one case of acquired resistance so far. In addition, other mechanisms of PARPi resistance involve modifications of the drug target, i.e., PARP1 conformation change to avoid PARP1 trapping—a phenomenon mentioned previously caused by PARPi drugs, although at present this has only been reported in pre-clinical BRCA1 mutant cancer models (Pettitt et al., 2018). Lastly, alterations in drug efflux system of the cell, like ABCB1 upregulation can decrease the concentration of PARP inhibitors in the cell and lead to resistance (Baxter et al., 2022).

Understanding PARPi or any other DDR inhibition drug resistance will reveal therapeutic opportunities to target newly emerging dependencies in adapted cancer cells. In recent years combination therapy has been proposed to overcome acquired PARPi resistance in clinical settings, such as combination of PARPi with topoisomerase I inhibitors, or with radiotherapy which is still being evaluated in clinical trials (Chabot et al., 2017; Bardia et al., 2024). Lately, immunotherapy has shown promising results in treatment of DDR deficient tumor cells as monotherapy or as part of combination therapy. For instance, immune checkpoint blockade, like PD-1/PDL1 inhibitors has shown exciting potential in colorectal cancer (CRC) with MMR deficiency and/or microsatellite instability (Ganesh et al., 2019). Moreover, initial clinical trials of PARPi and anti-PDL-1 antibody combination has shown improved response rates compared to monotherapies in breast and ovarian cancer patients (Li et al., 2019). This is probably due to higher mutation burden and accumulation of unrepaired DNA fragments in the DDR-deficient tumor cells which re-activates anti-tumor immune responses more effectively. Also, depending on the type of resistance mechanisms, tumor cells usually present specific neoantigens than can be targeted by immunotherapy (Dias et al., 2021).

## 5 Newly identified zinc finger proteins in DNA repair and human disease

In recent years, the importance of zinc-finger containing proteins for DNA damage repair has begun to be appreciated. There is now an expanding network of zinc-finger containing proteins with roles across multiple DNA repair pathways, although their mode of action is still poorly understood (Vilas et al., 2018; Singh and van Attikum, 2021). Despite recent advances, there are still hundreds of zinc-finger containing proteins which are yet to be characterized to understand their biological function.

Several labs have attempted to identify novel proteins with key functions in DNA damage repair using high content screens coupled with cDNA libraries. These approaches have identified many new proteins involved in DNA repair, including several zinc-finger containing proteins which have yet to be characterized (Izhar et al., 2015; Martinez-Pastor et al., 2021). These studies have helped to shape our current understanding that zinc-finger proteins have many functions, beyond their traditionally appreciated role in gene transcription.

Following up on initial observations from the Elledge lab, Haico van Attikum’s laboratory has identified a critical role for ZNF384 in classical non-homologous end-joining (cNHEJ). Indeed, ZNF384 was rapidly recruited to sites of DNA damage within seconds and was shown to interact with Ku70/80 and was ultimately required for their efficient loading at DNA DSB sites and the subsequent loading of the critical cNHEJ effector proteins XRCC4 and Ligase 4 (Singh et al., 2021). These observations have potential clinical significance, given that rearrangements of ZNF384 are highly prevalent in acute lymphocytic leukemia (ALL) (Hirabayashi et al., 2017).

Another zinc finger protein, ZNF281, has also been shown to be important for DNA double-strand break repair. It has been reported that ZNF281 can directly influence the transcription of several DNA repair genes in response to DNA damage (Pieraccioli et al., 2016). More recent work has demonstrated that ZNF281 plays a more direct role in DNA double-strand break repair, by directly interacting with the key NHEJ protein XRCC4, whereby ZNF281 enhances XRCC4 recruitment to DNA double-strand breaks, facilitating efficient DNA repair via cNHEJ (Nicolai et al., 2020). These findings are potentially important clinically, given that ZNF281 has been implicated in several cancers and high expression of ZNF281 correlates with significantly poorer outcome in patients across at least 10 different cancer types (Hou et al., 2023).

Building upon an emerging role for zinc finger proteins in DNA double-strand break repair, ZNF432 was recently identified as a regulator of DNA end-resection. ZNF432 was recruited to DNA lesions in both a DNA and parylation dependent manner and was shown to be able to directly stimulate PARP1 activity (O’Sullivan et al., 2023). Interestingly, depletion of ZNF432 inhibited DNA PKCs phosphorylation, leading to an increase in Rad51 foci formation. Mechanistically, it is proposed that ZNF432 inhibits Exo-1 mediated DNA end-resection, conveying resistance to PARP inhibition. It will be interesting to see if such mechanisms of PARPi resistance mediated by the loss or mis regulation of zinc finger proteins are observed in the clinic in coming years.

Other innovative approaches have also identified key roles for zinc-finger proteins in DNA repair. For example, Lee Zou’s laboratory utilized an *Escherichia coli* protein biotin ligase, BirA^R118G^, fused with the DNA repair factor Rad18. The BirA^R118G^ mutant lacks substrate specificity and so biotinylates proteins promiscuously based on proximity. Using this approach, ZNF280C (ZPET) was identified as an important DNA double strand break repair factor, whereby it slows DNA end-resection by regulating the recruitment of the Mre11 nuclease to DNA double-strand break sites. Importantly, loss of ZNF280C resulted in increased end-resection and homologous recombination, which if observed in the clinic, could drive chemotherapy resistance (Moquin et al., 2019).

Another zinc-finger protein, ZNF830, has also been implicated in chemotherapy resistance. ZNF830 interacts directly with CtIP, regulating its recruitment to DNA damage sites. In a xenograft model of non-small cell lung cancer, depletion of ZNF830 was able to dramatically inhibit tumor growth in combination with treatment with the PARP inhibitor, Olaparib, although it remains to be seen whether targeting ZNF830 in established tumors will have similar effects (Chen et al., 2018).

There is therefore an emerging network of zinc finger proteins functioning in DNA double-strand break repair with potentially significant implications for therapy response in the clinic. More work is needed to understand the prognostic significance of these zinc-finger proteins, which may represent a novel method of stratifying patients for treatment with specific DNA damaging chemotherapeutic approaches.

## 6 Alternative lengthening of telomeres (ALT) pathway

Another genome maintenance pathway that is widely considered critical across many cancer types is the alternative lengthening of telomere (ALT) pathway. The ALT pathway is utilized in 10%–15% of cancers to counteract telomere attrition which occurs during the normal process of DNA replication (Cesare and Reddel, 2010). Without counteracting this attrition, telomere shortening would result in activation of the DNA damage response, resulting in growth arrest, i.e., cellular senescence. The ALT pathway is dependent on the homologous recombination pathway, and in recent years has become an attractive target for cancer therapy. ZNF827, a C2H2 zinc-finger domain containing protein was originally identified via proteomics analysis of telomeric chromatin and is required for the recruitment of the NuRD histone deacetylase complex to telomeric chromatin, whereby it prevents localization and binding of the shelterin complex and regulates the recruitment of HR proteins for activation of the alternative lengthening of telomere (ALT) pathway (Conomos et al., 2014).

More recently, ZNF827 has been shown to be critical for epithelial to mesenchymal transition (EMT) in several contexts including brain development and breast cancer metastasis. Mechanistically, ZNF827 reorchestrates the splicing landscape by regulating the recruitment of HDAC1 to distinct genomic loci which in turn impairs the progression of RNA Polymerase II, altering the splicing of key genes involved in EMT (Sahu et al., 2022). Most recently, the role of ZNF827 for genome integrity was further defined. ZNF827 was shown to bind to single stranded DNA and associates indirectly with the single strand binding protein complex, RPA. Importantly, ZNF827 is recruited to sites of DNA replication stress, and is able to activate the ATR-Chk1 signaling axis. Depletion of ZNF827 was shown to impair homologous recombination mediated repair and sensitized cancer cells to treatment with the topoisomerase I inhibitor topotecan, thereby identifying ZNF827 as a potential new therapeutic target (Yang et al., 2024).

## 7 Zinc finger proteins as DDR inhibition targets

**FIGURE 2:**
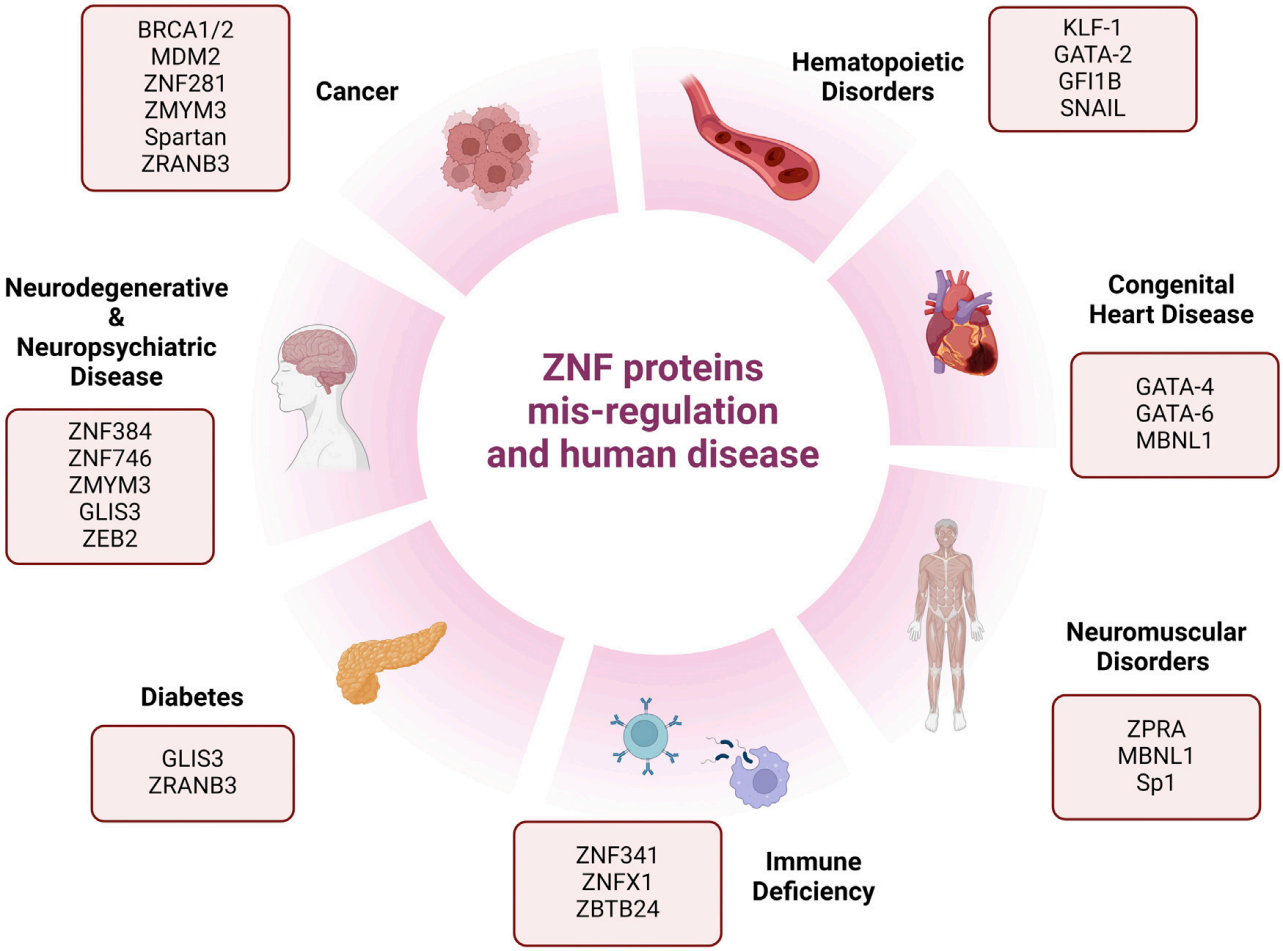
Schematic representation of different human phenotypes associated with mis-regulation of zinc-finger proteins. This figure represents reported human diseases and deficiencies associated with zinc-finger protein mis-regulation.

Considering the challenges and complex mechanisms of PARPi resistance, there is a clinical need to discover new targetable DNA repair proteins that can be used as monotherapy or in combination with other DDR drugs to exploit vulnerabilities in tumor cells that have acquired resistance to targeted therapies. In recent years, numerous studies have investigated and revealed the extended roles of ZNFs in human health, as summarized in Figure 2, beyond cancer (as oncogenes or tumor suppressors), but also in neurodegenerative diseases like Parkinson (ZNF746), Spinal muscular atrophy or SMA (ZPR1), diabetes (GLIS3), congenital heart disease (GATA4), and many more (Figure 2).

For instance, Leung et al. (2017) identified ZMYM3, a new DDR protein with ZNF domains which is required for BRCA1 localization to DNA damage sites. This work indicated that knocking out ZMYM3 results in HR deficiency and PARPi sensitivity in tumor cells. Moreover, a very recent study reported ZMYM3 deleterious variants in an X-linked neurodevelopmental disorder (NDD), introducing ZMYM3 as a neurodevelopmental disorder (NDD) gene (Hiatt et al., 2023). Spartan is a PHD-containing ZNF, characterized by Centore and colleagues as a reader of ubiquitinated PCNA and is needed for DDR proteins accumulation after UV exposure. PCNA (proliferating cell nuclear antigen) is an essential component of DNA replication and repair machinery which becomes ubiquitylated after disrupted replication in human cells, forming distinct molecular marks for post-replication DNA repair. Spartan also regulates Rad18, another PHD ZNF and the writer of PCNA ubiquitination (Centore et al., 2012). ZRANB3 is another example of a multi-functional ZNF. This endonuclease is redirected to the sites of ongoing replication forks as well as stalled and stressed replication forks. Remarkably, this gene also plays a significant role in genetic susceptibility to diabetes 2 in people of African ancestry (Ciccia et al., 2012; Adeyemo et al., 2019). Therefore, investigating novel ZNFs and elucidating their specific role in DNA repair pathways could help us to identify a myriad of novel therapeutic targets.

## 8 Future perspectives

In spite of increasing recognition, there are still many ZNF proteins that remain uncharacterized. Understanding the biological function of these zinc finger proteins will be critical to further our understanding of several human diseases including many different types of cancer. For example, exploring synergistic effects of ZNF inhibition with other DDR drugs like PARPi or even immune checkpoint blockade may present a promising avenue in therapeutic interventions to overcome chemoresistance. On the other hand, for developmental disease or in the case of transcription factor ZNFs, which have long been considered undruggable due to the lack of defined ligand binding sites, new approaches that promote selective protein degradation might be applicable. These novel therapeutic tools categorized as molecular glue degraders and PROTACs (proteolysis targeting chimeras), primarily function by recruiting an E3 ligase to a target protein to trigger its ubiquitylation and natural proteosome-mediated degradation within cells (Tsai et al., 2024). Excitingly, targeted degradation of IKZF1 and IKZF3- ZNF transcription factors essential for hematopoiesis, utilizing thalidomide as a PROTAC is FDA approved for use in multiple myeloma (Fischer et al., 2014). Therefore, as technologies improve, the possibility of therapeutically targeting many of these uncharacterized zinc finger proteins, could 1 day become a very realistic prospect. In conclusion, the multifaceted nature of ZNFs makes them a compelling target for future research and therapeutic development. By unlocking the potential of these proteins, we can pave the way for innovative strategies to overcome challenges like acquired drug resistance and develop more effective treatments for a range of human pathologies.
